# L-LOP/LOPP for the treatment of canine gastrointestinal/hepatosplenic lymphoma

**DOI:** 10.3389/fvets.2024.1373180

**Published:** 2024-05-23

**Authors:** Yu Ying Lai, Rodrigo dos Santos Horta, Angel Almendros, Patrick W. Y. Ha, Antonio Giuliano

**Affiliations:** ^1^Department of Veterinary Clinical Sciences, Jockey Club College of Veterinary Medicine, City University of Hong Kong, Kowloon, Hong Kong SAR, China; ^2^Department of Veterinary Medicine and Surgery, Veterinary School, Universidade Federal de Minas Gerais, Belo Horizonte, Brazil; ^3^CityU Veterinary Medical Centre, City University of Hong Kong, Kowloon, Hong Kong SAR, China

**Keywords:** canine, lymphoma, gastrointestinal, hepatosplenic, chemotherapy, LOP, LOPP

## Abstract

Canine gastrointestinal (GI) and hepatosplenic (HS) high-grade (large cell) lymphomas are uncommon forms of canine lymphomas, with a very poor response to chemotherapy and a very poor prognosis. Currently, there are no established effective chemotherapy protocols for canine GI/HS lymphomas. This case series aimed to retrospectively evaluate the efficacy of lomustine-based protocols L-LOP (L-asparaginase, lomustine, vincristine, and prednisolone) and L-LOPP (with the addition of procarbazine) for treatment of canine GI/HS lymphomas. Medical records of dogs with cytologically or histologically diagnosed lymphoma at CityU Veterinary Medical Centre from 2019 to 2022 were retrospectively reviewed. The L-LOP/LOPP treatment protocol was well tolerated with rare severe adverse events. Median progression-free survival for GI and HS lymphoma was 56 days (range, 10–274 days) and 57 days (range 8–135 days) respectively; while median survival time for GI and HS lymphoma was 93 days (range 10–325 days) and 210 days (range 8–240 days) respectively.

## 1 Introduction

Lymphoma is one of the most common canine malignancies contributing to 85% of canine hematopoietic cancers (1). The classification of lymphoma is mainly based on the anatomical locations, grade, and immunophenotype. The most common form of high-grade lymphoma is the multicentric form that affects mainly the peripheral lymph nodes. Lymphoma of the gastrointestinal (GI) tract affects the GI tract and mesenteric lymph nodes, and hepatosplenic (HS) lymphoma is characterized by infiltration within the hepatic and splenic sinusoids (2–4). Lymphoma is categorized histologically into intermediate to high- or low-grade, as well as B- or T-cell immunophenotype, based on the Revised European American Lymphoma/World Health Organization (REAL/WHO) system for lymphoid neoplasms (5). This system also includes a cytological classification of lymphoma into large-, intermediate- or small-cells. Grading can only be established by histopathology, however, in the clinical setting, tru-cut biopsies are rarely collected in dogs with internal organ abnormalities and fine needle aspirates (FNAs) are preferred as a minimally invasive procedure carrying a low risk of complications. In the most common type of lymphoma, cell size and appearance obtained by cytology are often associated with clinical behavior, with large cells and small cells often associated with clinically aggressive and indolent progression of the lymphoma, respectively (6).

Gastrointestinal and hepatosplenic lymphomas are rare in dogs. Gastrointestinal lymphoma comprises 5–7% of all canine lymphomas, while there were only a few cases of HS lymphoma previously reported (2–4, 7–10). Upon presentation, dogs with either GI or HS large cell lymphoma show aspecific clinical signs, including inappetence, vomiting, diarrhea, lethargy, and weight loss (2, 4, 11). Both forms of lymphoma share similar biological behavior: both exhibit aggressive clinical progression, respond poorly to chemotherapy, and have a poor prognosis despite treatment (median survival time post-diagnosis ranging from 13 days to only a few months) (3, 4, 8, 9, 11–15).

The recommended treatment modality for canine lymphoma is chemotherapy (1). However, there are currently no effective and established treatment protocols for canine GI/HS lymphomas. COP^1^/CHOP^2^-based multiagent chemotherapy protocols, which are the gold standard treatment for canine multicentric lymphoma, have been administered as the first-line treatment for canine GI/HS lymphomas with very limited efficacy (4, 13, 14).

LOP^3^/LOPP^4^-based multiagent chemotherapy protocols have been used commonly as rescue protocols for relapsed canine lymphoma, with an overall response rate and complete remission of 52–61% and 27–36% reported for LOPP protocol (16, 17). Recent studies assessing the use of LOP/LOPP-based protocols as first-line treatment for multicentric high-grade canine T-cell lymphomas have shown a longer median survival time than other retrospective studies using CHOP-based protocols (18, 19). Another study assessing the efficacy of continuous L-asparaginase as first-line treatment for large cell GI lymphoma in dogs has shown promising results, suggesting L-asparaginase as a reasonable option to be used in combination with other chemotherapeutic agents as first-line therapy (15). Owing to these findings, the use of L-LOP^5^/LOPP^6^-based multiagent chemotherapy protocols for the treatment of canine GI/HS lymphomas warrant further investigation.

This retrospective study aimed to evaluate the efficacy and safety of L-LOP/LOPP-based chemotherapy protocols as first-, second-, and third-line treatment for canine GI/HS lymphomas. An additional objective was to describe signalment and clinicopathologic features for dogs with GI/HS lymphomas.

## 2 Materials and methods

### 2.1 Case selection

Electronic medical records were retrospectively reviewed to identify dogs presented with high-grade (large cell) GI and HS lymphoma to a single referral center in Hong Kong (CityU Veterinary Medical Centre), from 2019 to 2022. Dogs that at presentation had predominantly involvement of the gastrointestinal tract and mesenteric lymph nodes without significant peripheral lymphadenomegaly were considered primary GI lymphoma, while dogs with predominantly affected liver and spleen without significant involvement of the peripheral lymph nodes or other organs were classified as HS lymphoma. Case selection was limited to dogs with cytologically or histologically confirmed large cell/ high-grade lymphoma treated with (L-)LOP/LOPP-based chemotherapy protocols (either LPP, LOP or LOPP, which includes lomustine, prednisolone, procarbazine, vincristine, plus/minus L-asparaginase).

### 2.2 Medical records review

Data collected from the medical histories included signalment (age, sex, neuter status, and breed), clinical signs at initial presentation, date of diagnosis, body weight at diagnosis, hematology and serum biochemistry including total serum calcium results, three views thoracic radiographs and abdominal ultrasonography, methods used for confirmation of diagnosis, methods and results of immunophenotyping, anatomic locations of the lymphoma, and the date and cause of death.

Details of the treatment protocols used (surgery, chemotherapy, or their combinations) were retrieved, including the type and dosage of each chemotherapeutic drug used as first-, second- and third-line treatment. First-line treatment was defined as the planned chemotherapy protocol for each patient at the beginning of the treatment course while second- or third-line treatment involved a change to another chemotherapy protocol or agent based on the attending veterinarian’s decision (after deeming the patient non-responsive to the previous treatment, following lymphoma relapse, or due to severe adverse events (AEs) related to the previously used protocol).

Response to treatment, including clinical response and objective response, were retrospectively analyzed according to the RECIST (Response Evaluation Criteria for Solid Tumors) criteria for canine lymphoma (20). Adverse events after chemotherapy were categorized and graded according to the Veterinary Cooperative Oncology Group–Common Terminology Criteria for Adverse Events version 2 guidelines (21). Staging was performed for all patients with three views thoracic radiographs and abdominal ultrasound. Assessment of response was achieved by repeated abdominal ultrasound examination and measurement of the mass/lesions when present. Clinical improvement was judged by physical examination and owner interview at each revisit. All dogs were managed and followed up at the same institution from the diagnosis to the euthanasia or death of the patient. Details of the chemotherapy protocol can be found in Table 1.

**Table 1:** Median and range of doses of chemotherapeutic drugs administered as part of the L-LOP/LOPP protocol 21-day cycle.

Abbreviation	Drug	Median dose (range)
L-	L-asparaginase	10,000 IU/m2 (5000–13,000) Day 1
L	Lomustine	50 mg/m2 (30–60)- Day 7 every 3 weeks
O	Vincristine	0.5 mg/m2 (0.35–0.75) Day 2 (weekly)
P	Procarbazine	50 mg/m2 (35–65) day 1 for 10/15 days
P	Prednisolone	0.83 mg/kg daily (0.18–1.79)

### 2.3 Statistical analysis

Median survival time (MST) and progression-free survival (PFS) were calculated separately for dogs with gastrointestinal lymphoma and hepatosplenic lymphoma, using the Kaplan–Meier analysis method. Survival time was defined as the time from initial diagnosis to death from any cause. Dogs alive at the end of the study or lost to follow-up were censored. Progression free survival was defined as the time from confirmed diagnosis until disease progression, disease relapse, or death. For all analyses, a *p*-value of ≤0.05 was considered statistically significant. Statistical analyses were performed using standard softwares SPSS, v. 29, IBM Corp, and GrphPadPrism v. 6.02.

## 3 Results

### 3.1 Signalment

Fourteen dogs were retrieved from the clinic database, seven with GI lymphoma and seven with HS lymphoma. All dogs received treatment at CityU Veterinary Medical Centre. Data of dogs with GI/HS lymphoma were presented in Tables 2, 3, respectively.

**Table 2:** Data on 7 dogs with gastrointestinal lymphoma.

Case no.	Signalment			Diagnostic method	Immuno-phenotype	Affected locations	Treatments	Chemotherapy protocol			Clinical Response^**^	Objective Response^‡^	PFS^^^ (d)	Survival time (d)
	Age (year)	Breed	Sex^*^					First-line	Second-line	Third-line				
1	13	Shih tzu	M	Cytology	NA^†^	Small intestine, mesenteric lymph nodes	Chemotherapy only	L-LPP	Doxorubicin/ epirubicin, cytarabine, l-asparaginase	None	+	CR	274	325
2	13	Jack russell terrier	MN	Cytology	NA	Small intestine	Chemotherapy only	L-LOPP	None	None	+	SD	18	18
3	13	Husky	MN	Cytology	NA	Small intestine, mesenteric and peripheral lymph nodes, liver, spleen, CNS^#^	Chemotherapy only	L-LOP	None	None	+	PR	37	37
4	7	Husky	FN	Histology	T-cell	Small intestine, mesenteric lymph nodes	Surgery and chemotherapy	LOPP	Doxorubicin	None	+	SD	94	124
5	4	English bulldog	M	Cytology	NA	Small intestine, stomach, mesenteric and peripheral lymph nodes, CNS	Chemotherapy only	L-LOP	None	None	−	PD	10	10
6	7	Shih tzu	MN	Cytology	T-cell	Small intestine, liver, spleen, mesenteric and peripheral lymph nodes	Chemotherapy only	L-LOP	None	None	+	PR	56	93
7	14	Shih tzu	FN	Cytology	NA	Small intestine, mesenteric lymph nodes	Chemotherapy only	L-LOPP	Modified L-CHOP	Cytarabine, chlorambucil, l-asparaginase	+	CR	78	113

^*^M = Male; MN = Male neutered; F=Female; FN=Female neutered.

^**^Clinical response was reported for the chemotherapy protocol(s) of interest (i.e., L-LPP or L-LOP or (L-)LOPP). + = improvement; − = no improvement.

^‡^Objective response was reported for the chemotherapy protocol(s) of interest (i.e., L-LPP or L-LOP or (L-)LOPP). CR = Complete response; PR = Partial response; SD=Stable disease, PD=Progressive disease.

^^^PFS was reported for the chemotherapy protocol(s) of interest (i.e., L-LPP or L-LOP or (L-) LOPP).

^†^NA = not available.

^#^CNS=Central nervous system.

**Table 3:** Data on 7 dogs with hepatosplenic lymphoma.

Case no.	Signalment			Diagnostic method	Immuno-phenotype	Affected locations	Treatments	Chemotherapy protocol			Clinical response^##^	Objective response^‡^	PFS^^^ (d)	Survival time (d)
	Age (year)	Breed	Sex^*^					First-line	Second-line	Third-line				
8	11	Cross breed	MN	Cytology	T-cell	Liver, spleen, mesenteric and peripheral lymph nodes	Chemotherapy only	L-COP	Doxorubicin	L-LPP, cytarabine	+	SD	59	110^^^^
9	13	Poodle	MN	Cytology	NA^†^	Liver, spleen, peripheral lymph nodes, eyes	Chemotherapy only	L-COP	LOP	None	+	SD	133	197^^^^
10	6	French bulldog	MN	Cytology	B-cell	Liver, spleen, small intestine, mesenteric and peripheral lymph nodes, central nervous system	Chemotherapy only	L-CHOP	LPP	Modified CHOP, cytarabine	+	PD	24	210
11	13	Corgi	MN	Cytology	NA	Liver, spleen	Chemotherapy only	Lomustine, l-asp^#^	None	None	−	PD	8	8
12	8	Shiba inu	MN	Cytology	T-cell	Liver, spleen, mesenteric lymph nodes	Chemotherapy only	L-LOP, leflunomide	Doxorubicin, leflunomide, l-asp	None	+	PD	57	Lost to follow-up
13	6	Cross breed	FN	Cytology	T-cell	Liver, spleen, mesenteric and peripheral lymph nodes, bone marrow, kidneys	Chemotherapy only	L-LOP	L-CHOP, cytarabine, chlorambucil	None	+	SD	135	240
14	12	Japanese spitz	MN	Cytology	NA	Liver, spleen, peripheral lymph nodes, skin	Chemotherapy only	LOP	Doxorubicin, l-asp	None	+	CR	57	64

^*^M = Male; MN = Male neutered; F=Female; FN=Female neutered.

^##^Clinical response was reported for the chemotherapy protocol(s) of interest (i.e., lomustine, (L-)LPP or (L-)LOP). + = improvement; − = no improvement.

^‡^Objective response was reported for the chemotherapy protocol(s) of interest (i.e., lomustine, (L-)LPP or (L-)LOP). CR = Complete response; PR = Partial response; SD=Stable disease, PD=Progressive disease.

^^^PFS was reported for the chemotherapy protocol(s) of interest (i.e., lomustine, (L-)LPP or (L-)LOP).

^^^^Non-lymphoma-related death.

^#^l-asp = L-asparaginase.

The median age at the time of diagnosis was 13 years (range 4–14 years). There were 5 males (5/7 [71%]), 3 castrated and 2 sexually intact; and 2 females (2/7 [29%]), both spayed. The median weight at initial presentation was 7.1 kg (range 5.4–29.4 kg).

Dogs with HS lymphoma consisted of 6 breeds, namely Poodle (1/7 [14%]), French Bulldog (1 [14%]), Corgi (1 [14%]), Shiba Inu (1 [14%]) and Japanese Spitz (1 [14%]). There were also two mixed-breed dogs (29%). The median age at the time of diagnosis was 11 years (range 6–13 years). There were 6 males (6/7 [86%]), all castrated; and one spayed female (1/7 [14%]). The median weight at initial presentation was 13.4 kg (range 7.18–19.2 kg).

### 3.2 Clinical and ultrasound imaging findings

All dogs were classified into the substage b and showed different clinical signs at presentation. The most common clinical sign for dogs with GI lymphoma included diarrhea (7/7 [100%]), anorexia (7 [100%]), lethargy (5 [71%]), vomiting (5 [71%]), and weight loss (4 [57%]). On physical examination, 2 dogs (29%) were presented with painful abdomen while 2 other dogs had subjectively distended abdomen, but no significant ascites. Common findings on abdominal ultrasound were small intestinal wall thickening with loss of layering and/or small intestinal mass (7/7 [100%]), mesenteric lymph node(s) enlargement (6 [86%]), peritonitis (4 [57%]), mild splenomegaly (3 [43%]), mild hepatomegaly (2 [29%]) and stomach wall thickening (2 [29%]). Most dogs (5/7 [71%]) were presented with focal disease on abdominal ultrasound, including focal wall thickening with loss of wall layering of a small intestinal segment or stomach, or a hypoechoic irregularly vascularized small intestinal/mesenteric mass. Often coupled with the focal lesion was regional peritonitis (hyperechoic peritoneum or mesentery plus/minus a small amount of anechoic abdominal effusion in the area of the affected intestine) and enlarged hypoechoic mesenteric lymph nodes. Dogs with diffuse disease on abdominal ultrasound (2/7 [29%]) were presented with generalized wall thickening of the small intestines or stomach plus/minus layering loss and marked abdominal lymphadenopathy (multiple mesenteric lymph nodes enlarged with hypoechoic texture).

For dogs with HS lymphoma, the most common clinical signs were anorexia (6/7 [86%]), lethargy (6 [86%]), weight loss (4 [57%]), diarrhea (4 [57%]), and vomiting (4 [57%]). On physical examination, most dogs were presented with distended abdomen due to organomegaly (4/7 [57%]), while 2 other dogs (29%) had painful abdomen. Common findings on abdominal ultrasound included hepatomegaly (7/7 [100%]), splenomegaly (6 [86%]), and mesenteric lymph node(s) enlargement (5 [71%]). One dog (14%) showed tracheobronchial lymph node(s) enlargement on thoracic radiography. In dogs with hepatomegaly on abdominal ultrasound, the most common presentation was generalized increased echogenicity and coarse architecture of the liver (4 out of 7 cases [57%]), while others presented as normal echogenicity, hypoechoic, or diffused infiltration by mixed echogenic multi-nodular masses within the hepatic parenchyma. The enlarged spleen mostly appeared diffusely heterogeneous on ultrasound, either with mottled echotexture and multiple miliary to coalescing hypoechoic nodules, or diffusely effaced by mixed echogenic multi-nodular mass lesions.

### 3.3 Laboratory abnormalities

Both hematology and serum biochemistry including total calcium tests were conducted in all cases at the time of initial diagnosis and during every weekly recheck, but serum ionized calcium and bone marrow evaluation were not performed in any of the patients. Dogs with GI lymphoma were commonly presented with mild to moderate anemia (5/7 [71%]), mild to moderate hypoalbuminemia (5 [71%]), mildly increased liver parameters (2 [29%] – median value of increased ALT of 302.5 U/L and ALP of 1106.5 U/L), mild thrombocytopenia (2 [29%]), mild neutrophilia (2 [29%]), and mild hypoglycemia (2 [29%]). For dogs with HS lymphoma, mildly increased liver parameters (6/7 [86%] – median value of increased ALT of 354.5 U/L and ALP of 1403.6 U/L), mild thrombocytopenia (5 [71%]), mild anemia (4 [57%]), and mild hypoalbuminemia (3 [43%]) were the most commonly reported findings.

### 3.4 Diagnosis and immunophenotype

Diagnosis of GI lymphoma was achieved mainly via cytology (6/7 [86%]) through ultrasound-guided FNAs of the small intestinal lesion and/or mesenteric lymph node(s). No FNAs of liver and spleen were obtained in those patients without significant abnormalities of the liver and spleen on ultrasound. One remaining dog was diagnosed by histology (Table 2, case 4) through exploratory laparotomy with resection and anastomosis of a grossly abnormal section of the small intestine. Immunophenotyping was performed for 2 dogs by immunocytochemistry (*n* = 1) and immunohistochemistry (*n* = 1), with both cases testing positive for T-cell CD3.

Diagnosis of all 7 cases of HS lymphoma was confirmed by cytology, through ultrasound-guided fine needle aspiration of the liver and spleen. Immunophenotyping was performed for 4 dogs by immunocytochemistry: 3/4 (75%) tested positive for T-cell CD3, while 1/4 (25%) tested positive for B-cell Pax-5.

### 3.5 Treatment

The majority of dogs with GI lymphoma (6/7 [86%]) received chemotherapy as the sole treatment, while 1 dog (Table 2, case 4) underwent surgical resection of the affected small intestinal segment followed by chemotherapy. All the chemotherapy protocols used as first-line treatment in GI lymphoma were LOP/LOPP based (either LPP, LOP or LOPP plus/minus L-asparaginase, as reported in Table 2). Rescue treatments were administered in 2 dogs (Table 2, case 1 and 4), whereas 1 dog received up to three rescue treatments (Table 2, case 7).

All dogs diagnosed with HS lymphoma were treated with chemotherapy only. Four out of 7 dogs (57%) were administered LOP/LOPP-based protocols as first-line treatment (either lomustine as a single agent or LOP plus/minus L-asparaginase, as reported in Table 3). LOP/LOPP-based protocols were used as second-line treatment in 2 cases (Table 3, case 9 and 10) and as third-line treatment in 1 case (Table 3, case 8). The median values and ranges of doses of each drug included in the L-LOP/LOPP protocol for the treatment of both gastrointestinal and hepatosplenic lymphoma (i.e., l-asparaginase, lomustine, vincristine, procarbazine, and prednisolone) are reported in Table 1.

### 3.6 Outcomes

#### 3.6.1 Survival time and progression-free survival (PFS)

**Figure 1:**
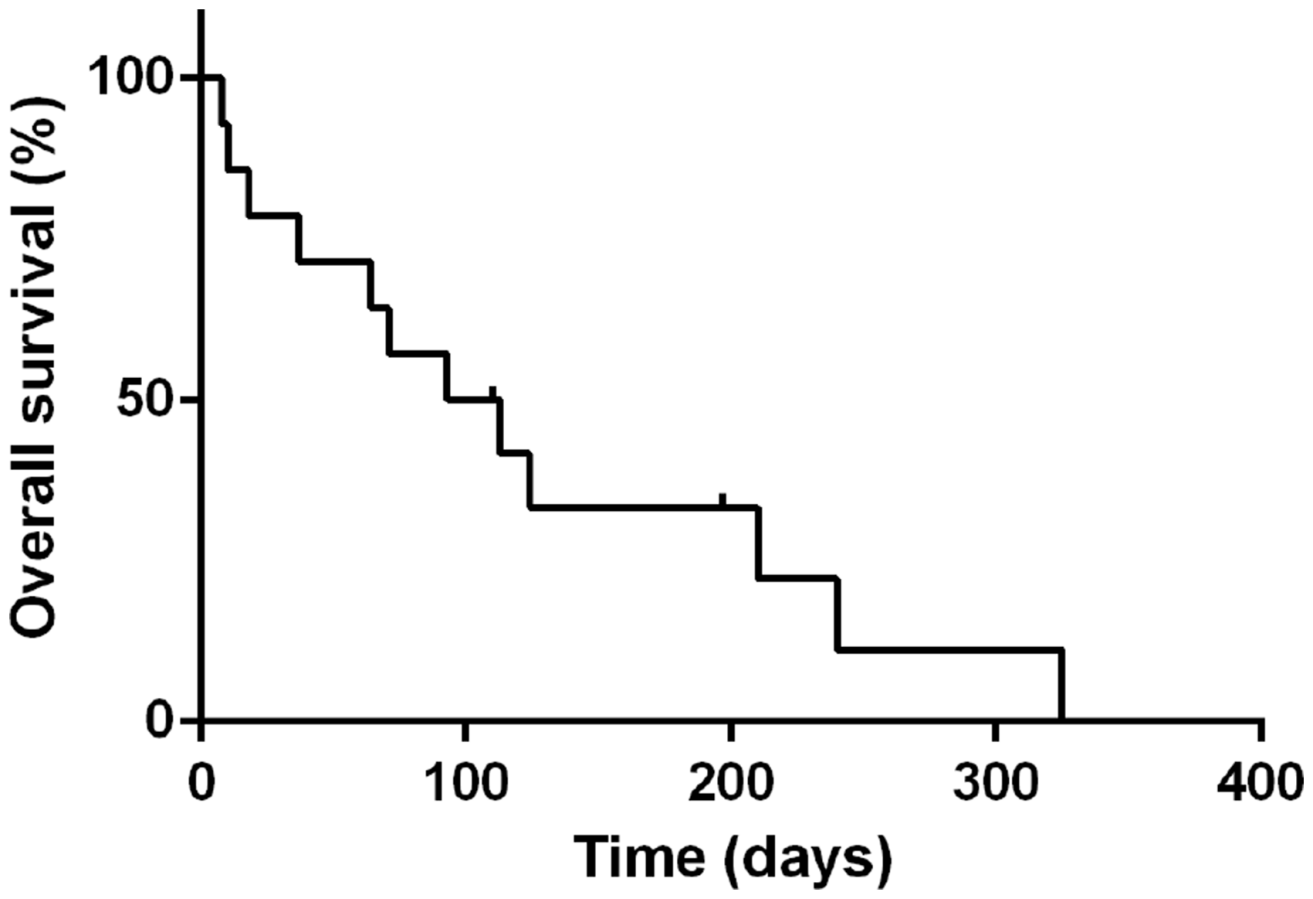
Median survival time of all 14 dogs treated with L-LOP/P. Dots represent censored patients.

The MST and PFS for all the 14 cases treated with the (L-)LOP/LOPP were 103 days and 57 days, respectively (Figure 1).

All seven dogs with GI lymphoma and 4 out of 7 dogs (57%) with HS lymphoma had died or were euthanized due to the lymphoma by the end of the study. Among the three remaining dogs with HS lymphoma, one was lost to follow-up, one died of pneumonia and sepsis, and one died of renal failure.

The MST and PFS for dogs with GI treated with (L-)LOP/LOPP-based chemotherapy protocols were 93 days (range 10–325 days); whereas the MST for dogs with HS lymphoma was 210 days (range 8–240 days).

The PFS for dogs with GI and HS lymphoma were 56 days (range 10–274 days) and 57 days (range 8–135 days) respectively.

#### 3.6.2 Response to treatment and adverse events

Of all the dogs with GI lymphoma that received chemotherapy as the sole treatment and were treated with LOP/LOPP as a first-line treatment, 5/6 dogs (83%) showed clinical improvement after starting the treatment, such as improved appetite, increased activity and weight gain. The dog that was surgically treated followed by first-line chemotherapy with LOPP (Table 2, case 4) also showed clinical improvement. As assessed by the RECIST criteria for lymphoma in dogs, 2/7 dogs (29%) showed complete response, 2/7 dogs (29%) showed partial response, 2/7 dogs (29%) showed stable disease, and 1/7 dog (14%) showed progressive disease after initial chemotherapy treatment (Table 2) (20).

For dogs with HS lymphoma that received LOP/LOPP-based chemotherapy treatments as first-, second-, or third-line treatment, 6/7 (86%) showed clinical improvement. Responses to LOP/LOPP chemotherapy were: 1/7 (14%) showed complete response, 3/7 (43%) showed stable disease, and 3/7 (43%) showed progressive disease (Table 3). The MST of patients who received L-LOP/LOPP-based first-line treatment was 64 days, (ranging from 8 to 240 days) while patients who received non-L-LOP/LOPP-based first-line treatment was 210 days (ranging from 110 to 210 days).

The main AE observed in 14 dogs after receiving L-LOP/LOPP-based chemotherapy, either as first-line treatments or rescue protocols, were reported in Table 4. Gastrointestinal adverse events (AEs) were reported in 79% of cases (11/14), which included hyporexia, vomiting and diarrhea. Both neutropenia and anemia in 71% of cases (10/14). Most adverse events observed were mild (Grade I or Grade II), and no Grade V adverse events were recorded.

**Table 4:** Incidence of adverse events of different severity after L-LOP/LOPP chemotherapy.

Category	Grade I	Grade II	Grade III	Grade IV	Total incidence
Gastrointestinal tract	5	6	0	0	79% (11/14)
Hematologic					
Neutropenia	2	2	3	3	71% (10/14)
Thrombocytopenia	3	2	0	3	57% (8/14)
Anemia	1	4	3	2	71% (10/14)
Hepatotoxicity	2	6	1	0	64% (9/14)

## 4 Discussion

A high-grade GI lymphoma is an aggressive form of lymphoma that carries a poor response to chemotherapy and a very poor prognosis. Similar to other studies, the findings of the present study showed a poor survival time with a median PFS of 56 days (range 10–274 days) and an MST of 93 days (range 10–325 days). Literature reports with CHOP-based protocols had MSTs of 60 days and 77 days, comparable with the MST of patients receiving L-LOP/LOPP-based chemotherapy in the present study (13, 14). In the other two studies, single-agent lomustine treatment (MST 144 days) and continuous L-asparaginase treatment (MST 147 days) had longer survival times than the current study (13, 15). From our and other previous small studies, it is unclear if lomustine-based protocols are superior or similarly effective to CHOP protocol. Larger prospective studies are necessary to have a definitive answer on what is the best chemotherapy treatment protocol, if any, for GI lymphoma in dogs.

In our study, the median PFS (57 days, range 8–135 days) and MST (210 days, range 8–240 days) for dogs with HS lymphoma were numerically much longer than in previous literature, which reported survival times ranging from 1 to 24 days with a single case exception surviving up to 196 days (4, 8, 9). A possible explanation is that in previously reported cases of HS lymphomas, only 2 out of 9 cases received chemotherapy, whereas the others showed rapid clinical deterioration and hence did not receive any treatment. However, in another study of dogs treated with CHOP-based protocol for presumed primary hepatic lymphoma an MST of 64 days was achieved (22).

The commonly reported clinical signs of GI and HS lymphomas in the present study (anorexia, lethargy, weight loss, diarrhea, and vomiting) coincide with previous studies (2, 4, 11, 13). these non-specific clinical signs with insidious onset may delay the confirmation of diagnosis, causing the patients to be presented with an advanced disease state resulting in a poorer prognosis.

The majority of GI and HS lymphomas are of T-cell origin (4, 8–13, 23). The present study was unable to exhibit a trend of immunophenotype for GI lymphoma, as most cases (5/7 cases) did not undergo immunophenotyping. In dogs with HS lymphoma, T-cell lymphoma appeared to be the predominant immunophenotype (3 cases of T-cell origin, 1 case of B-cell origin). Although there are only scarce reports of HS lymphoma in dogs it is likely to arise from splenic cytotoxic γδ-T-cells, which represents a specific syndrome in people known as hepatosplenic gamma-delta T-cell lymphoma (4, 8, 9). Nevertheless, primary or secondary HS large B-cell lymphomas are also reported in humans and may also occur in dogs (24, 25). Lack of immunolabeling for both T- and B-cell markers may also occur as a result of loss of T/B-cell antigen receptor complex expression (4). T-cell high-grade canine multicentric lymphomas have been associated with more aggressive biological behavior and poorer response to chemotherapy compared to the B-cell phenotype (26–28). However, there is no known prognostic significance for T and B immunophenotypes in GI or HS lymphoma.

L-LOP/LOPP-based chemotherapy exhibited an acceptable level of toxicity in the present study as most adverse events recorded were mild, and were all transient (i.e., patients recovered after reducing the dose or increasing the dosing intervals of chemotherapeutic drugs). In addition, the adverse events observed (e.g., diarrhea, vomiting) may not be entirely attributable to the drugs but may be due to the presence of the lymphoma or a combination of the above, which often represents a confounding factor, especially in retrospective studies. Lomustine-based treatment carries a risk of drug-induced hepatotoxicity (29) and the choice of administering a lomustine-based protocol in HS lymphoma could increase the risk of hepatotoxicity. Vincristine is also metabolized mainly in the liver and liver impairment could decrease the clearance of the drug and increase AEs (30). However, all the dogs were started on L-asparaginase and prednisolone followed by vincristine injection before the liver parameters improved and were considered acceptable. Only then, lomustine was administered. Both the doses of lomustine and vincristine were also reduced if some degree of liver impairment was suspected. The degree of dose reduction was established at the discretion of the clinician based on clinical assessment and the result of liver parameters.

There are a few limitations to this study. The retrospective nature of the study and the small number of cases for both groups is one of the most important limitations. The chemotherapeutic drugs used, dosages, and dosing intervals were not standardized. Histopathology confirmation and immunohistochemistry were performed in only one case; however, this is standard procedure in clinical practice where most large-cell/high-grade lymphomas are diagnosed by cytology, especially if involving internal organs. The lack of histopathology confirmation may result in the accidental inclusion of cases of other round-cell neoplasms with comparable survival, although this is quite unlikely. With canine GI/HS lymphomas being rare diseases, the sample size (*n* = 14) of the present study was small, resulting in low statistical power.

In the current study, the use of L-LOP/LOPP-based chemotherapy protocols for the treatment of canine GI/HS lymphomas has shown clinical improvement in most cases, comparable survival times with other chemotherapy protocols including CHOP, and well-tolerated AEs in patients, thus making it a viable treatment option which warrants further investigations. Future prospective studies are needed to better assess the efficacy of L-LOP/LOPP in GI and HS lymphoma in dogs.

## Data availability statement

The original contributions presented in the study are included in the article/supplementary material, further inquiries can be directed to the corresponding author.

## Ethics statement

The study has been approved by the animal research ethics committee of CityU, application n. ASTA-00000016.

## Author contributions

YL: Data curation, Formal analysis, Writing – original draft. RH: Writing – review & editing. AA: Writing – review & editing. PH: Data curation, Writing – review & editing. AG: Conceptualization, Data curation, Investigation, Methodology, Supervision, Writing – review & editing.

## Footnotes

^1^COP: cyclophosphamide, vincristine, prednisolone.

^2^CHOP: cyclophosphamide, doxorubicin, vincristine, prednisolone.

^3^LOP: lomustine, vincristine, prednisolone.

^4^LOPP: lomustine, vincristine, procarbazine, prednisolone.

^5^L-LOP: LOP with the addition of l-asparaginase.

^6^L-LOPP: LOPP with the addition of l-asparaginase.
